# A Model of Induction of Cerebellar Long-Term Depression Including RKIP Inactivation of Raf and MEK

**DOI:** 10.3389/fnmol.2017.00019

**Published:** 2017-02-06

**Authors:** Iain Hepburn, Anant Jain, Himanshu Gangal, Yukio Yamamoto, Keiko Tanaka-Yamamoto, Erik De Schutter

**Affiliations:** ^1^Computational Neuroscience Unit, Okinawa Institute of Science and TechnologyOkinawa, Japan; ^2^Theoretical Neurobiology, University of AntwerpAntwerp, Belgium; ^3^Department of Neurobiology, Northwestern UniversityEvanston, IL, USA; ^4^Center for Functional Connectomics, Korea Institute of Science and TechnologySeoul, South Korea

**Keywords:** long-term depression, Purkinje cell, dendritic spine, molecular modeling, stochastic simulation, cerebellum

## Abstract

We report an updated stochastic model of cerebellar Long Term Depression (LTD) with improved realism. Firstly, we verify experimentally that dissociation of Raf kinase inhibitor protein (RKIP) from Mitogen-activated protein kinase kinase (MEK) is required for cerebellar LTD and add this interaction to an earlier published model, along with the known requirement of dissociation of RKIP from Raf kinase. We update Ca^2+^ dynamics as a constant-rate influx, which captures experimental input profiles accurately. We improve α-amino-3-hydroxy-5-methyl-4 isoxazolepropionic acid (AMPA) receptor interactions by adding phosphorylation and dephosphorylation of AMPA receptors when bound to glutamate receptor interacting protein (GRIP). The updated model is tuned to reproduce experimental Ca^2+^ peak vs. LTD amplitude curves at four different Ca^2+^ pulse durations as closely as possible. We find that the updated model is generally more robust with these changes, yet we still observe some sensitivity of LTD induction to copy number of the key signaling molecule Protein kinase C (PKC). We predict natural variability in this number by stochastic diffusion may influence the probabilistic LTD response to Ca^2+^ input in Purkinje cell spines and propose this as an extra source of stochasticity that may be important also in other signaling systems.

## Introduction

**Figure 1:**
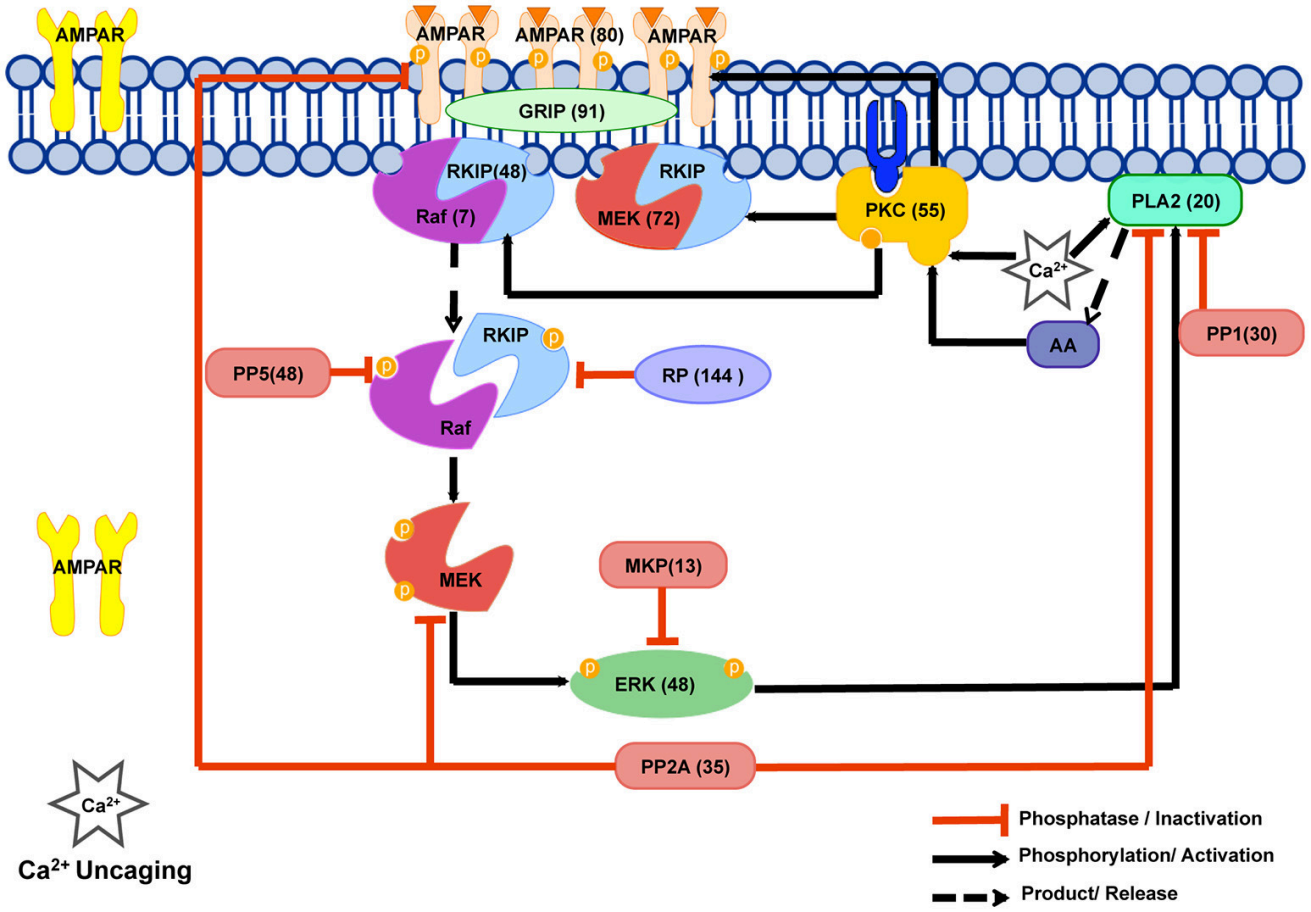
**The Cerebellar Long Term Depression molecular network in LTD_STEPS 2.1**. The phosphorylation and dephosphorylation pathways in our model. This network is activated by increase in intracellular Ca^2+^ and the model has been tuned to Ca^2+^ uncaging experiments. Numbers in brackets give the initial number of molecules in the spine for each simulation and correspond to the estimated number of molecules located in one spine of a Purkinje cell dendrite of volume 0.08 μm^3^ where a concentration of 1 μM corresponds to 48 molecules. PKC phosphorylates AMPAR and is responsible for its reduction from the surface leading eventually to LTD. Our model includes a MAPkinase feedback loop, which involves Raf, MEK, ERK, cPLA_2_ and AA molecules. Transiently activated by Ca^2+^ initially, PKC activity is maintained by this feedback loop. PP2A dephosphorylates AMPAR, thus counteracting PKC activity on AMPAR, and also suppresses feedback loop activity. Raf regulation has been improved by the addition of the RKIP mechanism in the current version of the model, and MEK-RKIP interaction has also been included. This scheme was illustrated using the Motifolio PPT Drawing Toolkits (Motifolio Inc, MD, USA).

Cerebellar long-term depression (LTD) is a decrease in the efficacy of synaptic transmission from parallel fibers (PFs) to Purkinje cells (PCs) (Ito, 2001). A transient increase in postsynaptic Ca^2+^ concentration is crucial for the induction of LTD, leading to activation of many molecular species that are implicated in synaptic plasticity (Tanaka and Augustine, 2008; Figure 1). The molecular processes result in phosphorylation of AMPA-type glutamate receptors (AMPARs) by Ca^2+^-dependent protein kinase C (PKC) and their subsequent removal from the surface of the PF-PC synapse (Ito, 2001; Evans, 2007; Gallimore et al., 2016). Based on experimental evidence, the mechanism that is responsible for maintaining PKC activation after the Ca^2+^ stimulus has finished is a Ca^2+^-activated positive feedback loop involving a specific mitogen-activated protein kinase, the extracellular signal-regulated kinase (ERK; Bhalla and Iyengar, 1999; Tanaka and Augustine, 2008; Antunes and De Schutter, 2012). In this pathway, PKC activation leads to activation of Raf by a process that involves phosphorylation of RKIP (Yamamoto et al., 2012). Raf activates Mitogen-activated protein kinase kinase (MEK), which activates ERK. Activated ERK then activates cytosolic phospholipase A_2_ (cPLA_2_), which produces arachidonic acid (AA), which activates PKC (O'Flaherty et al., 2001) completing the loop. Within the network, Protein phosphatase 2 (PP2A) plays a duel role; it dephosphorylates MEK (Sontag et al., 1993) suppressing feedback loop activity, and dephosphorylates AMPARs (Launey et al., 2004) counteracting AMPAR internalization. Due to the small size of PC dendritic spines, stochastic simulation of the network is necessary and an initial stochastic model predicted that single synapses exist in one of two discrete stable states—LTD or non-LTD—with no clear threshold of response to the input Ca^2+^ signal (Antunes and De Schutter, 2012); features which are absent with deterministic modeling.

In this study we present an update to the 2012 stochastic LTD model (Antunes and De Schutter, 2012) with several new components. For convenience, we term the 2012 model “LTD_STEPS 1.0” and the updated models presented in this paper “LTD_STEPS 2.0” and “LTD_STEPS 2.1.” Recent experiment evidence shows the role of Raf kinase inhibitor protein (RKIP) in regulating Raf (Corbit et al., 2003; Yamamoto et al., 2012). RKIP binds to Raf-1 making an RKIP-Raf complex (Figure 1). Activation of PKC by a transient increase in Ca^2+^ leads to phosphorylation of RKIP bound to Raf, which results in release of Raf from the complex. Based on this evidence we improve LTD_STEPS 1.0 by adding more realistic reactions contributing to Raf activation. It's important to note at this point that in our model we do not distinguish between the three distinct Raf isoforms: Raf-1 (or c-Raf), B-Raf and A-Raf (Matallanas et al., 2011). There is evidence for expression of at least two different isoforms in Purkinje cells, Raf-1, and B-Raf, and with difference subcellular locations (Morice et al., 1999). In our previous work we saw clear expression of Raf-1 in Purkinje cell dendrites (Yamamoto et al., 2012) and, based on these observations, we have largely focused on Raf-1 specific interactions when developing the LTD model. However, earlier reports have suggested B-Raf is also expressed in Purkinje cell dendrites (Morice et al., 1999), so we cannot rule out some role of B-Raf within the pathway. However, Raf-1 and B-Raf perform similar functions, both binding and phosphorylating MEK, although with different kinetics (Matallanas et al., 2011). RKIP is known to associate with both Raf-1 and B-Raf isoforms (Trakul et al., 2005) and has been shown to inhibit B-Raf activation in cancer cells (Park et al., 2005) as well as the strong interaction with Raf-1 previously mentioned. So due to the similar role for Raf-1 and B-Raf within the pathway, including activation of MEK and interaction with RKIP, and lack of conclusive experimental evidence for more detailed modeling, our approach is to include a generic Raf molecule in the feedback loop with overall activity tuned to match experimental data, and not to incorporate isoform-specific kinetics.

In addition to its interaction with Raf, RKIP has been reported to interfere with Mitogen-activated protein kinase kinase (MEK) phosphorylation by forming ternary complexes with MEK, resulting in the suppression of feedback loop activation (Yeung et al., 2000). To understand this significance in the context of cerebellar LTD we also investigate experimentally whether dissociation of RKIP from MEK is required for PKC activation of the ERK pathway, and make further improvements to our model based on these findings. We also improve the Ca^2+^ dynamics and adjust several parameters so that both the Ca^2+^ input signal and response of the model mimic experimental observations closely.

We compare LTD_STEPS 2.1 to LTD_STEPS 2.0 and LTD_STEPS 1.0 in terms of noise propagation and sensitivity, concluding that LTD_STEPS 2.1 appears to be generally more robust, yet with no adverse affect on LTD induction. As an initial practical application of LTD_STEPS 2.1, we test the influence of cytoplasmic PKC copy number—which may vary naturally within spines by effects such as stochastic diffusion and varying spine morphology—on LTD induction. Differing LTD induction probabilities are observed within the estimated natural range of cytoplasmic PKC, and we propose that dynamic variability in signaling molecules may act as an extra significant source of stochasticity in synaptic signaling pathways.

## Materials and methods

### Experimental procedures

All procedures involving mice were carried out in accordance with the recommendations of the Institutional Animal Care and Use Committee of Korea Institute of Science and Technology. The protocol was approved by the Institutional Animal Care and Use Committee of Korea Institute of Science and Technology.

#### Cloning experiments

Recombinant glutathione-S-transferase (GST) and GST-fused mutant of Raf kinase inhibitory protein (RKIP-TV-delRBD) were purified from *E. coli* using glutathione sepharose (Amersham Biosciences). RKIP-TV-delRBD possesses a single mutation of threonine residue 153 to valine (Corbit et al., 2003) and a deletion mutation of amino acids 77–108, which include a main site for its association with Raf-1 (Yeung et al., 2000). Purified proteins were dialyzed in HEPES buffered solution (20 mM HEPES, pH 7.2), concentrated by centrifugal filter devices (Millipore), and stored at −80°C.

#### Electrophysiology experiments

Chemicals were obtained from Sigma or Wako Pure Chemical Industries, unless otherwise specified. Whole-cell patch clamp recordings were made from Purkinje cells in sagittal slices (200 μm) of cerebella from 17- to 21-day-old mice of either sex. Slices were bathed in extracellular solution (ACSF) containing (in mM): 125 NaCl, 2.5 KCl, 1.3 MgCl_2_, 2 CaCl_2_, 1.25 NaH_2_PO_4_, 26 NaHCO_3_, 20 glucose, and 0.01 bicuculline methochloride (Tocris). Patch pipettes (resistance 5–6 MΩ) were filled with (in mM): 130 potassium gluconate, 2 NaCl, 4 MgCl_2_, 4 Na_2_-ATP, 0.4 Na-GTP, 20 HEPES (pH 7.2), 10 phosphocreatine and 0.25 EGTA. Recombinant proteins (1–2 mg/ml) were added into the internal solution along with dextran-conjugated fluorescein (Molecular Probes), to monitor the dialysis of intracellular solutions into Purkinje cells by a fluorescent microscope (Olympus BX61WI or Nikon Eclipse FN1). Excitatory postsynaptic currents (EPSCs) were evoked in Purkinje cells (holding potential of −70 mV) by activating parallel fibers (PFs) with a glass-stimulating electrode on the surface of the molecular layer (PF-EPSCs). PF-EPSCs were acquired and analyzed using pClamp software (Molecular Devices). To evoke LTD by electrical stimulation (PF&ΔV), PF stimuli were paired with Purkinje cell depolarization (+10 mV, 200 ms) 300 times at 1 Hz. Data were accepted if the series resistance changed <20%, input resistance was >80 MΩ, and holding current changed <10%.

Statistical differences were determined by the Student's *t*-test and analysis was performed with OriginPro 8.1 software.

#### Immunoblotting experiments

The inhibitory effect of RKIP-TV-delRBD on ERK phosphorylation was tested by immunoblotting, as described previously (Yamamoto et al., 2012). In brief, human embryonic kidney 293T (HEK293T) cells transfected with pEGFP-RKIP-WT or pEGFP-RKIP-TV-delRBD were treated with or without TPA (100 nM) for 5 min and lysed in lysis buffer containing the following: 150 mM NaCl, 50 mM Tris, pH 8, 1 mM EDTA, 0.2% NP-40 alternative (Calbiochem), 10% glycerol, 1X proteinase inhibitor cocktail, and 1X phosphatase inhibitor cocktail. The supernatants of cell lysates were subjected to SDS-PAGE analysis to detect phosphorylated ERK, ERK, and GFP by immunoblotting. Primary antibodies used were rabbit anti-ERK (Cell Signaling Technology), rabbit anti-phospho-ERK (Cell Signaling Technology) or mouse anti-GFP (Roche Applied Science). Secondary antibodies used were horseradish peroxidase-conjugated anti-mouse or anti-rabbit IgG (GE Healthcare) antibodies.

### Addition of RKIP reactions to LTD_STEPS 1.0

Due to lack of experimental evidence on specific mechanisms for Raf activation LTD_STEPS 1.0 contained an artificial Raf activation reaction (Antunes and De Schutter, 2012). *Raf-Act* molecule became activated by PKC, and then activated Raf. Recently, a role of RKIP in regulating Raf activation during cerebellar LTD has been proposed experimentally (Corbit et al., 2003; Yamamoto et al., 2012). According to these experimental results in Purkinje cells, RKIP inhibits the Raf activation of MEK by making complexes with Raf. Experimental characterization using high-resolution heteronuclear nuclear magnetic resonance spectroscopy (Odabaei et al., 2004) demonstrated that Raf-RKIP interaction is regulated mainly by the phosphorylation of RKIP at Ser-153. RKIP binds to Raf in the native form and when phosphorylated releases Raf. The Raf released from the complex may or may not be active. Experimental evidence suggests that PKC phosphorylates RKIP and dissociates the RKIP-Raf complex, usually releasing active Raf (Corbit et al., 2003).

To obtain the Raf-RKIP binding kinetic parameters, we analyzed a published mathematical model that explores the kinetic parameters for Raf-RKIP complex formation (Kwang-Hyun et al., 2003). This model includes a reaction in which artificial molecule RKIP phosphatase (RP) dephosphorylates phosphorylated RKIP. This model is also based on the old theory of Raf-RKIP complex dissociated by ERK, which is now generally refuted since the evidence of phosphorylation was based on the wrong pocket-binding site of RKIP previously established. Initially we replicated this model in STEPS and validated the implementation. We then modified this isolated model by removing the ERK phosphorylation reaction on RKIP. We added a cPLA_2_-AA-PKC loop to this isolated model, where ERK phosphorylates cPLA_2_, which activates PKC via AA (Supplementary Figure 1). In our new isolated model of the loop, PKC dissociates the Raf-RKIP reaction, with phosphorylation kinetics as that of ERK in the model. The artificial RP reaction was retained in this modified isolated model due to no experimental results for alternative RKIP dephosphorylation (although some studies suggests a fast de-phosphorylation of RKIP; Lorenz et al., 2003).

We added the isolated model to LTD_STEPS 1.0 and termed the new model LTD_STEPS 2.0. RKIP also forms complexes with MEK (Yeung et al., 2000) and thus we added the RKIP-MEK complex to LTD_STEPS 2.0. We used the same parameters for RKIP-MEK complex as for RKIP-Raf reactions. In our model and with the experimental evidence shown in Section Dissociation of RKIP from Raf and MEK is Required for Cerebellar LTD, the dissociation of both the complexes is by activated PKC. In LTD_STEPS 2.0, activated PKC transfers to the membrane. The dissociation of Raf-RKIP complex by active PKC is a surface reaction and then the phosphorylated Raf and phosphorylated RKIP are transported into the cytoplasm again for further activities. We increased the Raf concentration to 0.15 μM (up from 0.10 μM in LTD_STEPS 1.0) based on recent experimental measurements of its average concentration (Fujioka et al., 2006). In cell populations the RKIP concentration is known to be around 5–10 times higher than Raf (Corbit et al., 2003). In LTD_STEPS 2.0, we used a RKIP concentration of 1.0 μM.

We then added the interactions of PKC and PP2A with glutamate receptor interacting protein (GRIP)-bound AMPAR (GRIP_AMPAR) to the model, as described in Section Addition of PKC-GRIP_AMPAR and PP2A-GRIP_AMPAR Interactions Makes the Model More Robust and Reduces Fluctuations of ERK and cPLA_2_, and termed this model LTD_STEPS 2.1, so that the only difference in terms of molecular interactions between LTD_STEPS 2.0 and LTD_STEPS 2.1 is the inclusion of the GRIP_AMPAR interactions in LTD_STEPS 2.1. LTD_STEPS 2.1 was then tuned by making small modifications to capture dose-response curves accurately compared to experimental data, as described in Section Updated Ca^2+^ Dynamics Captures Experimental Input Ca^2+^ Profile and LTD Dose-Response Accurately. Apart from these changes mentioned, other binding parameters and concentrations were maintained from LTD_STEPS 1.0 (Antunes and De Schutter, 2012).

### Simulations

All simulations were run in software STEPS (Hepburn et al., 2012). The LTD models were run as stochastic well-mixed models.

Where computing the mean response of the bistable LTD model (such as for data shown in **Figures 4, 5**) 200 iterations of the model were run with different random number seeds, and mean calculated over those 200 iterations. The LTD response was measured as the proportion of synaptic AMPARs 50 min after Ca^2+^ stimulus relative to before stimulus.

The diffusion simulation of **Figure 7** was run on a tetrahedral mesh describing the dendritic morphology. This mesh consisted of a section of dendrite 20 μm length and diameter 1 μm with 20 spines of varying morphological properties (varying head volume, varying neck widths, and varying neck lengths) constrained by experimental observations (Harris and Stevens, 1988). The simulations were run for 1000 s and population of PKC molecules recorded in all spine heads every 0.01 s.

## Results

### Dissociation of RKIP from Raf and MEK is required for cerebellar LTD

**Figure 2:**
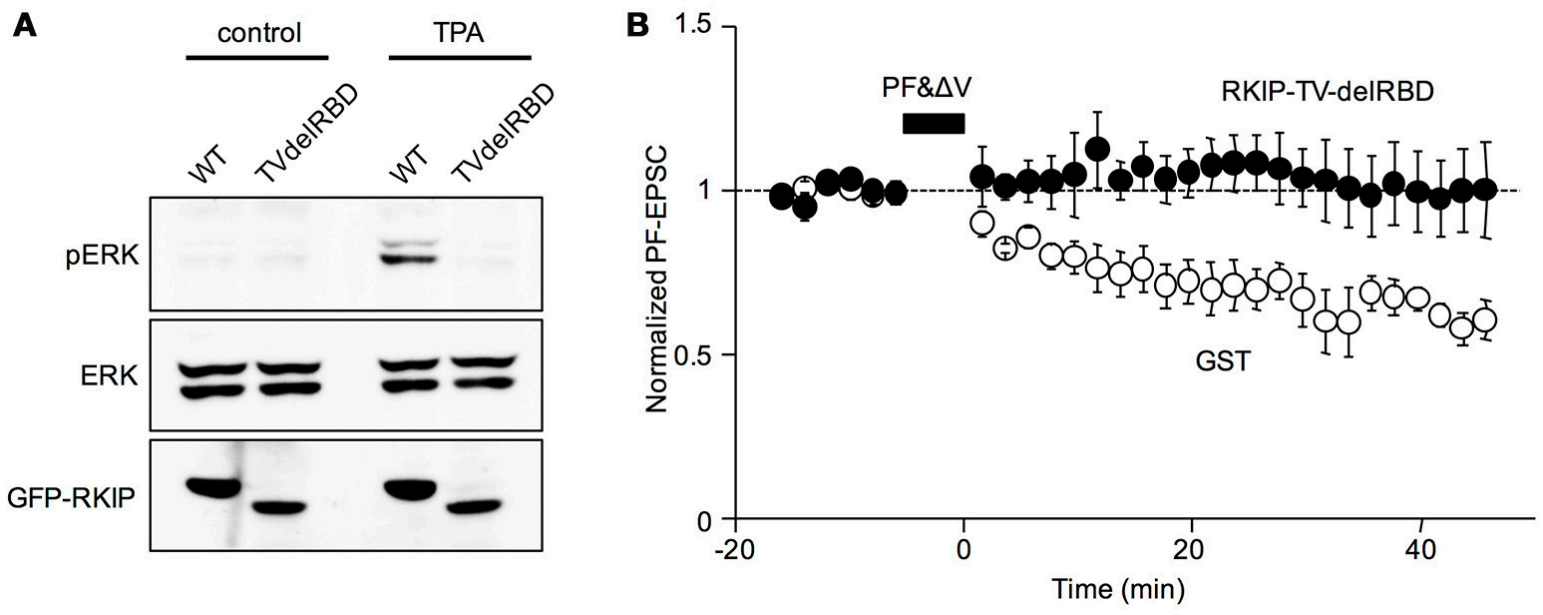
**The requirement of dissociation of RKIP from MEK for LTD. (A)** Immunoblot analysis of ERK phosphorylation in HEK293T cells expressing GFP fused RKIP-WT or RKIP-TV-delRBD. TPA (100 nM) was extracellularly applied for 5 min to activate PKC. **(B)** Time course of LTD induced by PF&ΔV in the presence of GST (*n* = 4, open circles) or RKIP-TV-delRBD (*n* = 5, filled circles). PF-EPSC amplitudes are normalized to their mean prestimulation level. Error bars represent SEM.

Recent experiment evidence shows the role of Raf kinase inhibitor protein (RKIP) in regulating Raf (Corbit et al., 2003; Yamamoto et al., 2012). While RKIP interacts with and inhibits Raf, PKC-dependent phosphorylation of RKIP at serine or threonine 153 causes dissociation of RKIP from Raf, allowing Raf to activate downstream kinases MEK and ERK, so that the main pathway of PKC activating ERK is thought to be mediated by the dissociation of RKIP from Raf (Corbit et al., 2003; Lorenz et al., 2003; Figure 1). However, because RKIP was also shown to directly interact with MEK (Yeung et al., 2000), the dissociation of RKIP from MEK seems to be also required for the pathway of PKC activating ERK. Our previous study demonstrated that while a non-phosphorylatable RKIP mutant (RKIP-TV) inhibited cerebellar LTD, deletion of the binding site for both Raf and MEK in RKIP-TV reversed this effect (Yamamoto et al., 2012). Biochemical analysis revealed the dissociation of RKIP from both Raf and MEK after LTD stimulation (Yamamoto et al., 2012). These results suggest that the dissociation of RKIP from not only Raf, but also MEK, following RKIP phosphorylation is required for LTD. In the current study, we confirmed the requirement of dissociation of RKIP from MEK for LTD by using another deletion mutant of non-phosphorylatable RKIP (RKIP-TV-delRBD), which lacks a main site for its association with Raf, yet includes a site binding with MEK (Yeung et al., 2000). The immunoblot analysis in HEK293T cells demonstrated that RKIP-TV-delRBD inhibited ERK phosphorylation triggered by TPA, a PKC activator (Figure 2A), confirming that RKIP-TV-delRBD interrupts ERK activation presumably through constitutive MEK binding. When control protein, GST, was introduced into Purkinje cells, the pairing electrical stimulation induced LTD (Figure 2B). On average, PF-EPSCs were depressed by 32.9 ± 5.2% (*n* = 4) at 20–40 min after the end of PF&ΔV. In contrast, introducing RKIP-TV-delRBD into Purkinje cells blocked LTD and the depression was significantly smaller (−7.2 ± 8.7%; *n* = 5; *p* < 0.01) than that in the presence of GST (Figure 2B). Because RKIP-TV-delRBD can be considered to interact with only MEK, this result supports that dissociation of RKIP from MEK following RKIP phosphorylation is also required for LTD.

### Updated Ca^2+^ dynamics captures experimental input Ca^2+^ profile and LTD dose-response accurately

**Figure 3:**
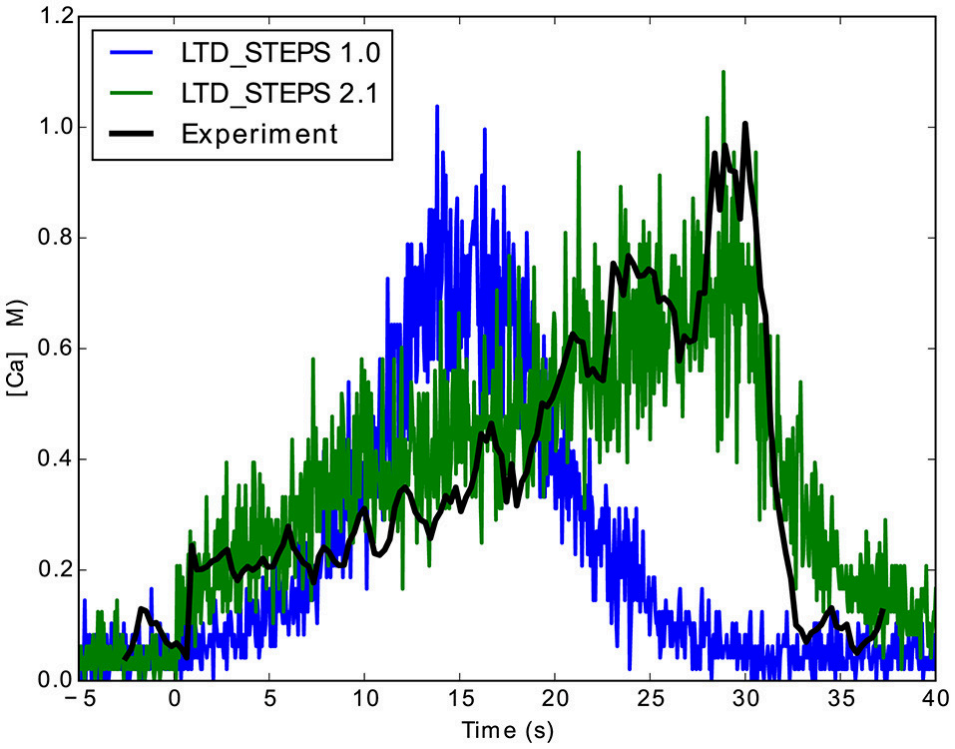
**Comparison between the measured Ca^2+^ response to the constant rate Ca^2+^pulse in LTD_STEPS 2.1 (green) and Gaussian Ca^2+^ pulse in LTD_STEPS 1.0 (blue) to experimental data (black) for a 30 s Ca^2+^ uncaging stimulus**. The simulated pulses have the same peak value as the uncaging stimulus.

LTD_STEPS 1.0 simulated changes in Ca^2+^ concentration due to Ca^2+^ uncaging with Gaussian-shaped Ca^2+^ stimuli of durations from 1 to 60 s (Antunes and De Schutter, 2012). We obtained Ca^2+^ concentration measurements of the original experimental Ca^2+^ pulses with durations of 0.5, 1, 15, and 30 s (Tanaka et al., 2007) and observed that they are skewed (Figure 3). Therefore, we fitted a constant-rate response to mimic the experimental Ca^2+^ uncaging dynamics more closely in LTD_STEPS 2.1 (Figure 3). The constant-rate response was modeled as a simple production reaction for the duration of the pulse:

(1) $$\overset{k}{\to }[Ca^{2+}]$$

where different rates, *k*, give varying peaks of the *Ca* pulse.

The experimentally measured degree of *LTD* induced by *Ca*^2+^ pulses of different amplitudes follows a sigmoidal relationship (Tanaka et al., 2007):

(2) $$LTD=LTD_{\max}\left(\frac{{\left[Ca^{2+}\right]}^{n_{Hill}}}{K_{1/2}^{n_{Hill}}+{\left[Ca^{2+}\right]}^{n_{Hill}}}\right)$$

**Figure 4:**
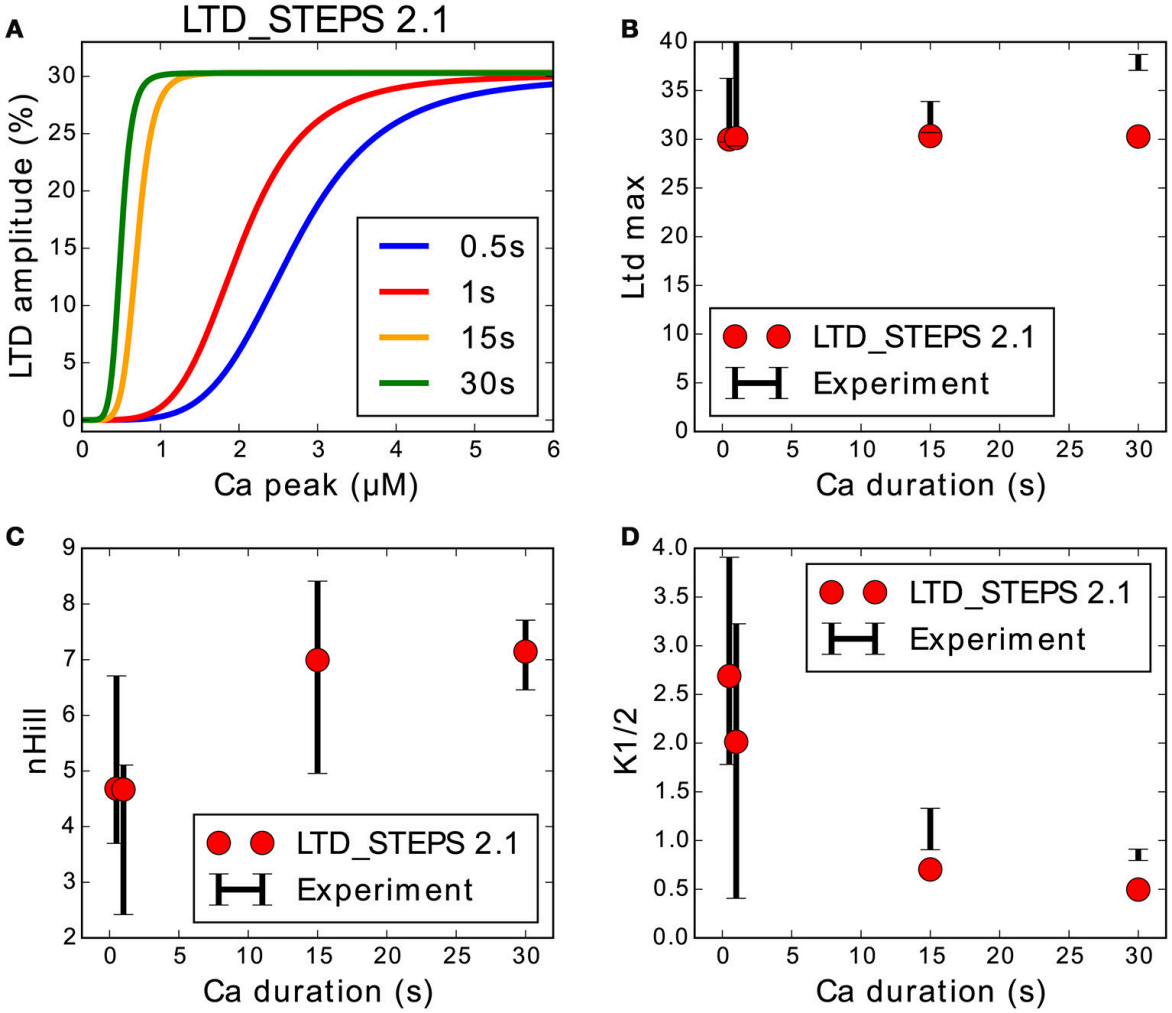
**Response of LTD_STEPS 2.1 compared to experimental Ca^2+^ uncaging data (Tanaka et al., 2007). (A)** LTD response to Ca^2+^ input of LTD_STEPS 2.1 for Ca^2+^ pulses of 0.5 s (blue), 1 s (red), 15 s (orange), and 30 s (green), which were the same pulse-widths as reported originally in uncaging experiments. **(B)** Maximum amplitude of LTD (%) of LTD_STEPS 2.1 (circles) and experiment (black error bars) at the four different Ca^2+^ pulse widths. **(C)** *n*_*Hill*_ (see Equation 1) of LTD_STEPS 2.1 (circles) and experiment (black error bars) at the four different Ca^2+^ pulse widths. **(D)** K_1/2_ (see Equation 1) of LTD_STEPS 2.1 (circles) and experiment (black error bars) at the four different Ca^2+^ pulse widths. In all cases for the fits to experimental data, the black error bars show one standard deviation as reported by the fitting software. Error bars are not shown for the simulation data because they were very small due to the large number of fitted points.

We refitted the output of the LTD_STEPS 2.1 model to the measurements of the original experimental data (Tanaka et al., 2007). We reduced the number of AMPARs at rest in the synapse to 80 (Masugi-Tokita et al., 2007). LTD magnitude was measured 30 min after input for different concentrations and pulse widths of Ca^2+^. Sigmoidal curves were obtained by the Scipy optimize curve_fit function for both the simulation data and experimental data. The parameters of the curves ([Hill coefficient (*n*_*Hill*_), the [Ca^2+^] required to achieve the half-maximum magnitude of LTD (*K*_1/2_), and maximum depression (*LTD*_max_)]) were measured from the optimized fits, and the model fine-tuned for best agreement between experimental observations and model behavior. The number of SERCA pumps in the model was reduced from LTD_STEPS 1.0 and the initial number of PKC and PP2A molecules adjusted to get the optimal fits. PKC was adjusted to 1.1 μM (55 molecules) from 1 μM in LTD_STEPS 1.0 and PP2A was reduced to 0.7 μM (35 molecules) from 1.5 μM. With these adjustments, the updated model shows realistic behavior quantitatively when compared to experimental measurements, with a perfect fit of *n*_*Hill*_ and a good approximation of *K*_1/2_ (Figure 4). These fits are better than those of LTD_STEPS 1.0, but it was not possible to fit both the experimentally observed *n*_*Hill*_ and *K*_1/2_ values perfectly over the entire range.

### Addition of PKC-GRIP_AMPAR and PP2A-GRIP_AMPAR interactions makes the model more robust and reduces fluctuations of ERK and cPLA_2_

**Figure 5:**
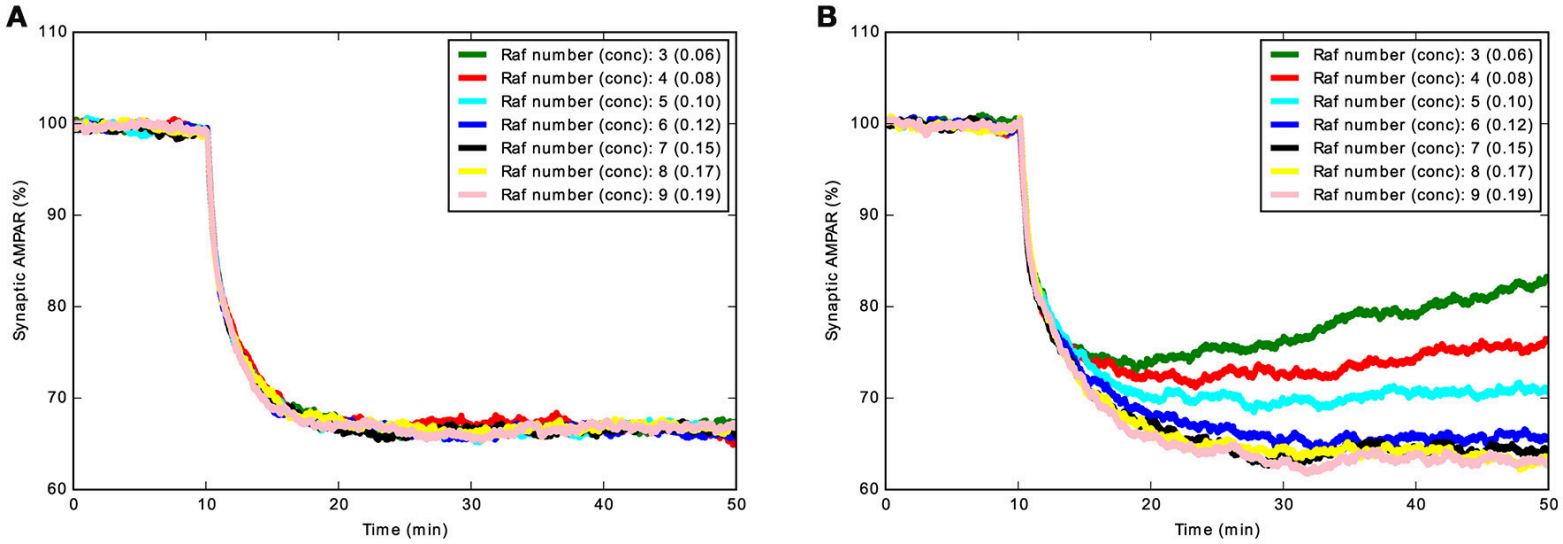
**Demonstration of increased robustness of LTD_STEPS 2.1: addition of AMPAR_GRIP interactions with PKC and PP2A decrease sensitivity to Raf copy number. (A)** Mean Synaptic AMPAR vs. time for LTD_STEPS 2.1 with varying Raf number (concentration given in brackets in units of μM) showing no sensitivity to Raf copy number within the tested 3–9 molecules range (normal conditions 7 molecules, 0.15 μM). **(B)** Mean Synaptic AMPAR vs. time for LTD_STEPS 2.0. This model shows strong sensitivity to Raf copy number within the same 3–9 molecules range. In both cases the Ca^2+^ input was 15 s duration with a peak of ~4 μM to give a 100% probability of LTD under normal conditions of Raf 0.15 μM (see Figure 4).

LTD_STEPS 1.0 did not allow phosphorylation by PKC and dephosphorylation by PP2A of synaptic AMPAR when bound to GRIP, only the small fraction of free AMPARs in the synapse could react with PKC and PP2A. The phosphorylation interactions with AMPAR bound to GRIP (GRIP_AMPAR) were added to the model LTD_STEPS 2.1 and the new behavior of this model compared to a version (LTD_STEPS 2.0) where these phosphorylation interactions are absent, but includes the addition of RKIP. Our first observation was that with the GRIP_AMPAR interactions the model appears more robust. This is demonstrated by ultrasensitivity of LTD induction to Raf copy number in LTD_STEPS 2.0, resulting in a lack of stable LTD induction when the number of Raf molecules was below 5 (5 was the number of Raf molecules in the LTD_STEPS 1.0 model; Figure 5). Such strong sensitivity to Raf copy number was not observed in the more robust model LTD_STEPS 2.1.

**Figure 6:**
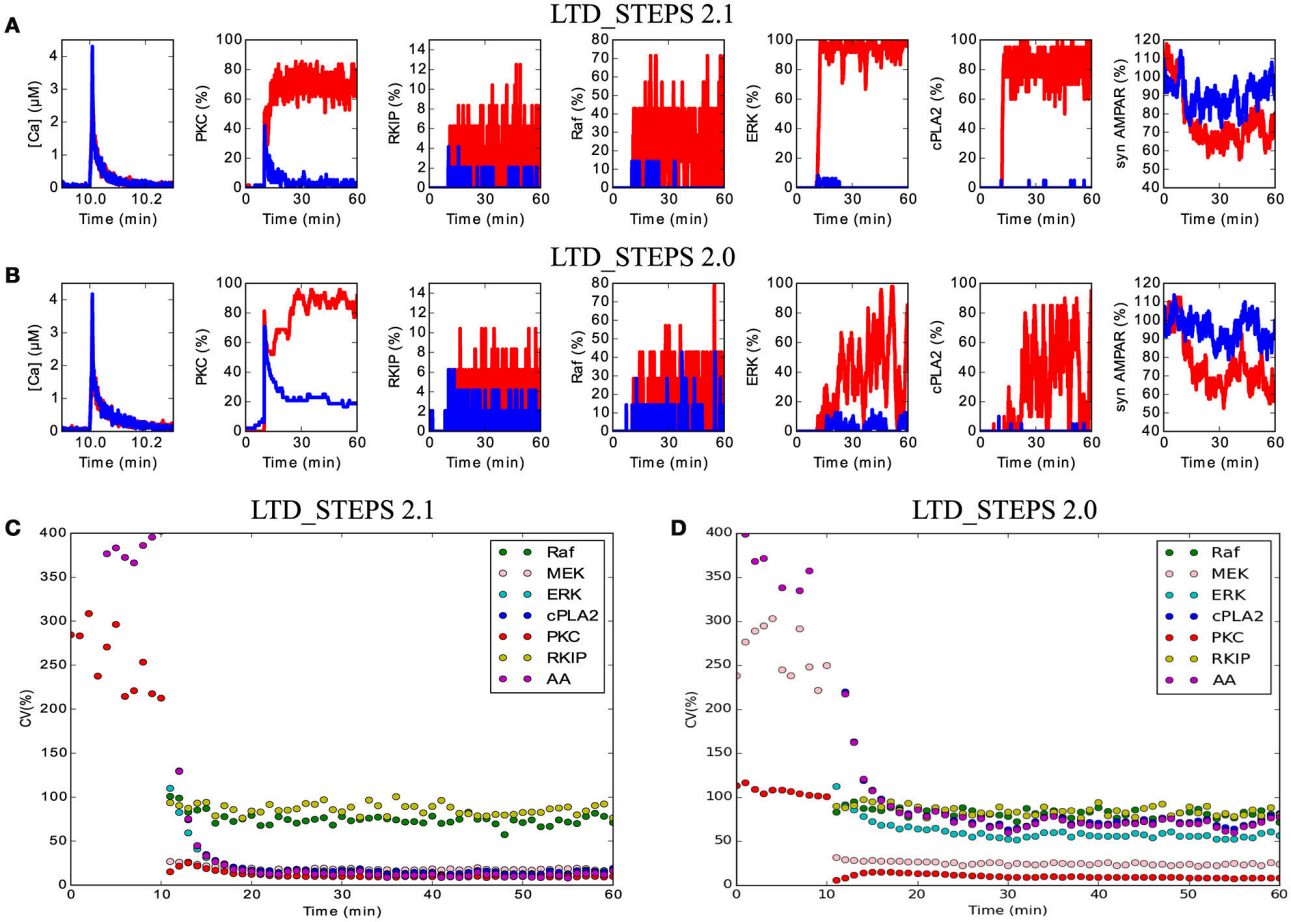
**Reduced noise within the network in LTD_STEPS 2.1. (A)** and **(B)** Activity in the molecular network over time of 1 h with short Ca^2+^ stimulus at 10 min for one example each of LTD (red traces) and non-LTD (blue traces) for models LTD_STEPS 2.1 **(A)** and LTD_STEPS 2.0 **(B)**. Note the stronger fluctuations in LTD_STEPS 2.0 LTD traces, particularly for ERK and cPLA_2_. Strong fluctuations of Raf and associated activated RKIP are observable for both models due to the low copy number of the Raf molecule. **(C)** Coefficient of variation for 7 different types of active molecule including some as shown in **(A)** for LTD_STEPS 2.1. Note, the observed weak fluctuations of ERK and cPLA_2_ result in a low CV. **(D)** The same CV measurements for LTD_STEPS 2.0. Note similar amplitude of CV for ERK, cPLA_2_ and AA in comparison to LTD_STEPS 1.0 (Antunes and De Schutter, 2012), which also omitted phosphorylation reactions with GRIP_AMPAR. For both models shown in **(C)** and **(D)** the Ca^2+^ input was longer in duration than for **(A)** and **(B)** at 15 s to give no expected failed runs of LTD (see Figure 4), so the CV measurements do not include any non-LTD traces.

With this change and other additions to the model, we tested one of the predictions of LTD_STEPS 1.0: strong fluctuations of activation of molecules in the positive feedback loop, in particular of ERK and the cPLA_2_, were suggested as important indicators of activity in the feedback loop (Antunes and De Schutter, 2012). However, with the addition of RKIP and its interactions with Raf and MEK, plus the phosphorylation and dephosphorylation of GRIP-bound AMPAR (model LTD_STEPS 2.1), the fluctuations of these molecules was severely diminished (Figures 6A,C) compared to the 2012 report (see Figures 4,5 of Antunes and De Schutter, 2012) and to the case where the interactions with GRIP-bound AMPAR are not included (model LTD_STEPS 2.0; Figures 6B,D). The loss of these fluctuations did not affect activity of the feedback loop, nor hinder induction of LTD (Figure 4).

### Stochastic diffusion from dendrite causes varying molecule population in spines

Dendritic spines cannot typically be thought of as isolated chemical compartments at relatively long timescales (Svoboda et al., 1996; Bloodgood and Sabatini, 2005), meaning that the inactive proteins that make up the initial state of the cytosolic signaling system may diffuse to and from the dendrite with the degree of dendritic coupling depending strongly on spine morphology (Bloodgood and Sabatini, 2005; Grunditz et al., 2008; Khan et al., 2012).

**Figure 7:**
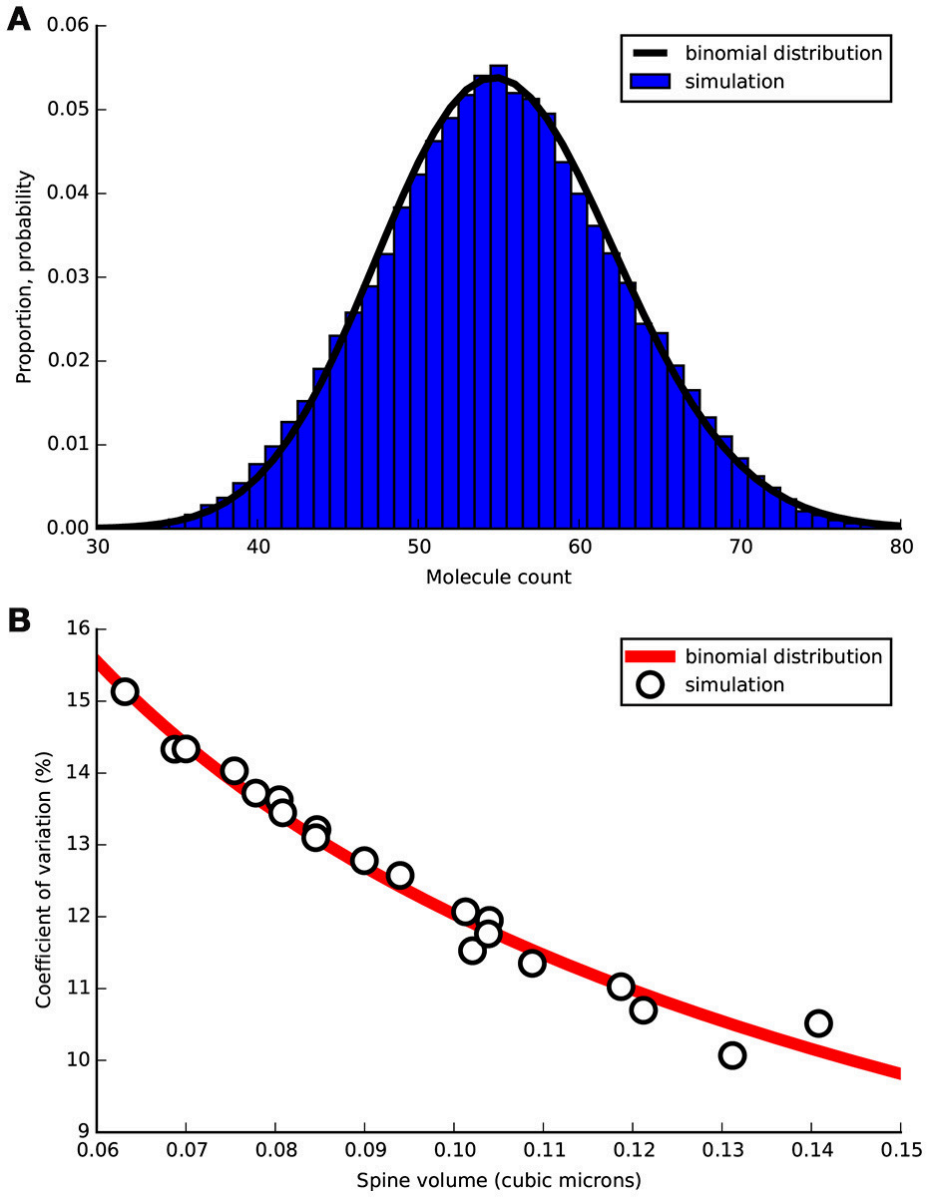
**Free PKC stochastic diffusion simulated in tetrahedral mesh representing a section of Purkinje cell dendrite 20 μm length containing 20 spines of varying morphological properties**. Simulation was run for 1000 s with PKC population sampled in each spine head every 0.01 s. **(A)** For one spine of volume 0.08 μm^3^ normalized measured distribution of molecules over the 1000 s sample time (blue bars) compared to binomial PDF (black line). **(B)** Coefficient of variation (CV) vs. spine head volume from simulation (circles) compared to expected CV by the binomial distribution (red line).

We constructed a diffusion model in realistic dendritic morphology to investigate how stochastic diffusion between dendrite and spine may affect the initial presence of cytosolic proteins in a spine before stimulus. We injected cytoplasmic PKC molecules at a concentration of 1.14 μM, the concentration used in LTD_STEPS 2.1 before LTD induction, and simulated diffusion with a rate of 5.45 μm^2^s^−1^ (Craske et al., 2005). Figure 7A shows the normalized distribution of the number of PKC molecules in a spine of volume 0.08 μm^3^ from simulation compared to the binomial distribution. Figure 7B compares the resulting measured coefficient of variation (CV) for all spines compared to the binomial distribution. As expected these results show that the binomial distribution is the correct description of expected molecule population per spine and so other morphological features play no important role on this long timescale. The relatively high CVs for these small volumes predicts a significant natural variability in molecule number per spine.

### PKC copy number affects probability of LTD induction

The results of Figure 7A demonstrates that PKC copy number, with a mean of 55, has a significant probability of being as low as ~40 and as high as ~70 by natural variation. We tested LTD_STEPS 2.1 under these two scenarios to investigate whether this range of variability in copy number could influence LTD induction.

**Figure 8:**
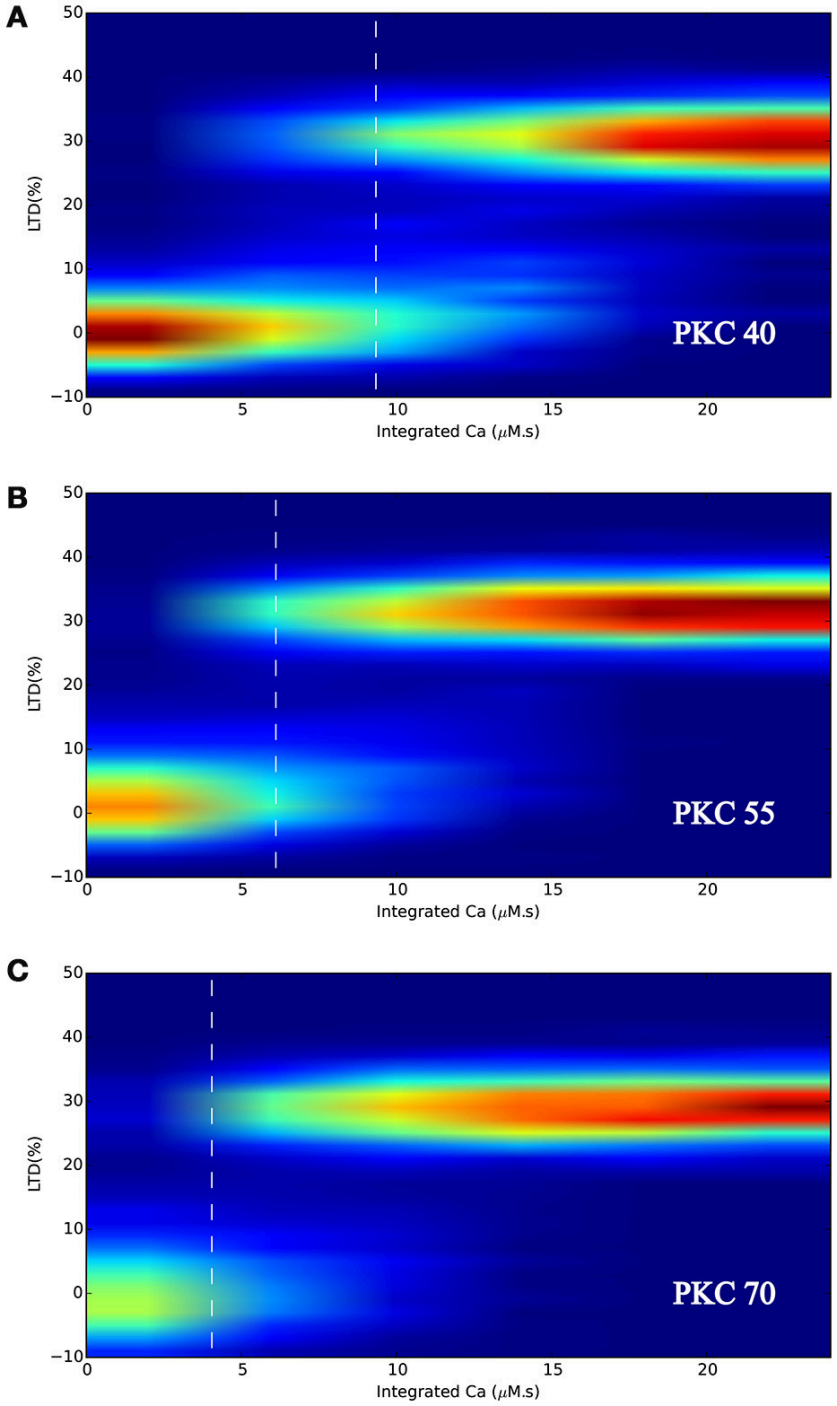
**LTD response heatmaps of LTD_STEPS 2.1 with varying initial PKC copy number of (A)** 40 molecules, **(B)** 55 molecules, and **(C)** 70 molecules. Ca^2+^ input to the model (e.g., Figure 3) is integrated for many different pulse durations and amplitudes and for the corresponding individual responses of the model, data is binned and normalized to produce the heatmaps. The vertical dashed white lines indicate the approximate position where 50% of the simulations can be classified as non-LTD responses (fluctuating around 0% LTD) and 50% can be classified as LTD responses (fluctuating around ~30% LTD).

Figure 8B shows the response of the model under normal conditions, i.e., with initial PKC copy number of 55, where the probabilistic bistable behavior already observed in LTD_STEPS 1.0 can clearly be seen. At 0 μM.s integrated Ca^2+^ input, all runs of the model respond by fluctuating around 0% LTD response, which we define as a non-LTD response corresponding to a physiological absence of spontaneous LTD. As integrated Ca^2+^ increases, some runs switch to the other bistable state, that of ~30% reduction in synaptic AMPARs, which we define as an LTD response. At some point (marked on the Figure with vertical dashed line) any individual run of the stochastic model has an equal probability of going to LTD or non-LTD, which is at ~6 μM.s integrated Ca^2+^ with normal PKC copy number (Figure 8B). As Ca^2+^ input increases further eventually all simulations give an LTD response.

Figures 8A,C show that varying PKC copy number shifted the probabilistic threshold for LTD induction: for 40 molecules of PKC (Figure 8A) the position of equal LTD or non-LTD induction was at integrated Ca^2+^ ~9 μM.s, whereas it was at integrated Ca^2+^ ~4 μM.s for 70 molecules of PKC (Figure 8C). Based on these results we predict that variation in cytoplasmic PKC copy number, which occurs by the diffusion of molecules within dendrite and spines (Figure 7A), may significantly influence the probability of LTD induction within a spine. We did not observe any other significant effect for varying PKC number in this range, such as a change in LTD amplitude.

## Discussion

We report an updated stochastic model of molecular LTD in parallel fiber-Purkinje cell synapses. The model is more realistic than LTD_STEPS 1.0 in terms of containing several new additions that are known to influence LTD induction and by more closely following experimental observations. We have shown experimentally that RKIP dissociation from MEK is necessary for LTD induction and we have added this interaction to the model as well as the known interactions of RKIP with Raf. The addition of PP2A and PKC interactions with AMPAR bound to GRIP appears to have increased the robustness of the model and reduced fluctuations of ERK and cPLA_2_ without, however, adversely affecting LTD induction. Adjustments to the Ca^2+^ input and output dynamics both improve Ca^2+^ profiles and capture LTD response curves accurately when compared to experiment.

When considering the initial state of the LTD system, the molecular population can be expected to show natural variation described by the binomial distribution. The binomial distribution is one of the most commonly applied probability distributions in biology and in recent years has found increasing use in cellular biology such as for controlling the probabilistic distribution and open probability of membrane channels (Hille, 2001) including those on single spines (Sabatini and Svoboda, 2000; Ribrault et al., 2011), molecular fluctuations in cell division (Kar et al., 2009), and it also has very wide use in genetics, but it has not up to now (to our knowledge) been applied specifically to describe intracellular signaling molecule populations within small subcellular volumes such as dendritic spines. In this study we find that this natural source of variability may significantly affect the probability of LTD induction. Specifically, we tested variation in PKC copy number, which was found to shift the LTD-Ca^2+^ response curves. We did not model dynamic changes in PKC copy number after stimulus for these simulations, which is no doubt a simplification but with some justification since it has been proposed that spines act dynamically to restrict diffusion through the neck after stimulus in order to retain second messengers and activated proteins in the spines (Bloodgood and Sabatini, 2005), although the degree of compartmentalization depends on the time course of inactivation (Harvey et al., 2008). Signaling systems can be expected to be more robust to the variability in some signaling molecules than others, however, based on our findings we expect generally the response of molecular signaling systems to have some sensitivity to the natural variability in copy numbers and propose this as an extra source of stochasticity for such systems.

## Author contributions

IH and AJ: Contributed to the LTD model, ran simulations, analyzed data and wrote the manuscript. HG: Contributed to the LTD model, ran simulations and analyzed data. YY: Performed molecular part of experiments and wrote part of the manuscript. KT: Performed electrophysiology part of experiments and wrote part of the manuscript. ED: Supervised the project and wrote the manuscript.

## Funding

This work was funded by the Okinawa Institute of Science and Technology Graduate University and the Korea Institute of Science and Technology Institutional Program (Project No. 2E26190).

### Conflict of interest statement

The authors declare that the research was conducted in the absence of any commercial or financial relationships that could be construed as a potential conflict of interest.

## Supplementary material

### Availability

The updated model, LTD_STEPS 2.1, is available at ModelDB: (http://senselab.med.yale.edu/ModelDB/showModel.cshtml?model=222732)

STEPS is available at http://steps.sourceforge.net
