# Supplementary material for: A Model of Induction of Cerebellar Long-Term Depression Including RKIP Inactivation of Raf and MEK
Source: Front Mol Neurosci. 2017 Feb 6;10:19. doi: 10.3389/fnmol.2017.00019 (PMC5292618; doi:10.3389/fnmol.2017.00019)
Supplement: Supplementary file 1 [file Image1.PDF]

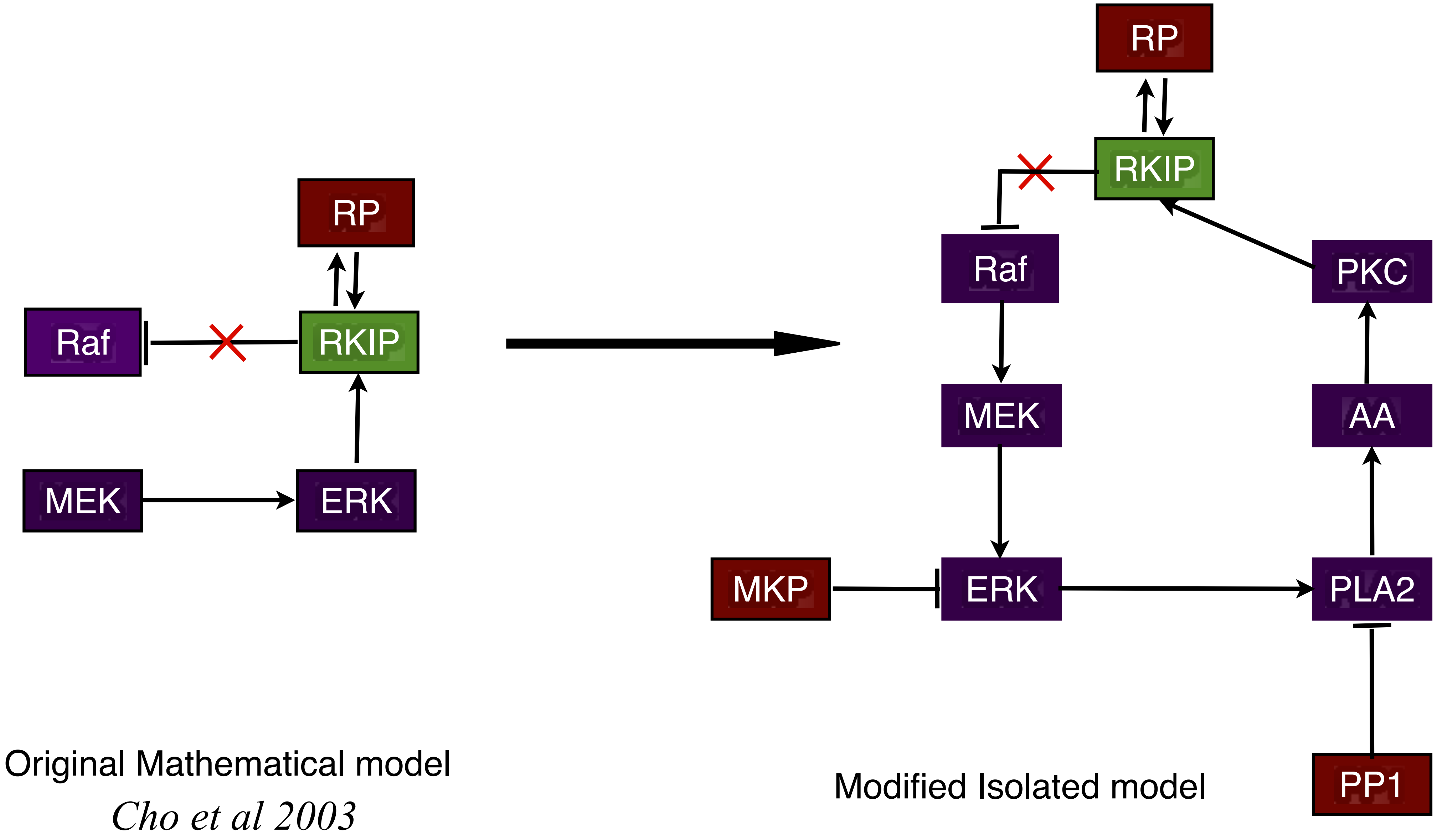

Figure S1: Development of the isolated model of Raf-RKIP kinetics
The original mathematical model (left panel) including Raf-RKIP binding kinetics. In the modified model (right panel) we added cPLA2, AA and PKC reactions completing the feedback loop. PP1 and MKP inhibitors were also added as dephosphorylation reactions. Phosphorylating molecules are represented in purple, dephosphorylating molecules in red and RKIP in green.
